# A dataset of fishes in and around Inle Lake, an ancient lake of Myanmar, with DNA barcoding, photo images and CT/3D models

**DOI:** 10.3897/BDJ.4.e10539

**Published:** 2016-11-09

**Authors:** Yuichi Kano, Prachya Musikasinthorn, Akihisa Iwata, Sein Tun, LKC Yun, Seint Seint Win, Shoko Matsui, Ryoichi Tabata, Takeshi Yamasaki, Katsutoshi Watanabe

**Affiliations:** ‡ Institute of Decision Science for Sustainable Society, Kyushu University, Fukuoka, Japan; § Department of Fishery Biology, Faculty of Fisheries, Kasetsart University, Chatuchak, Bangkok, Thailand; | Laboratory of Ecology and Environment, Division of Southeast Asian Area Studies, Graduate School of Asian and African Area Studies, Kyoto University, Kyoto, Japan; ¶ Inle Lake Wildlife Sanctuary, Nature and Wildlife Conservation Division, Forest Department, Ministry of Natural Resources and Environmental Conservation, the Republic of the Union of Myanmar, Nyaung Shwe, Myanmar; # Department of Zoology, Taunggyi University, Taunggyi, Myanmar; ¤ Laboratory of Animal Ecology, Graduate School of Science, Kyoto University, Kyoto, Japan; « Osaka Museum of Natural History, Osaka, Japan; » Yamashina Institute for Ornithology, Abiko, Japan

**Keywords:** Myanmar, Shan State, Inle Lake, freshwater fishes, endemic species, alien, GBIF, mitochondrial DNA, COI, CT scan, 3D model

## Abstract

**Background:**

Inle (Inlay) Lake, an ancient lake of Southeast Asia, is located at the eastern part of Myanmar, surrounded by the Shan Mountains. Detailed information on fish fauna in and around the lake has long been unknown, although its outstanding endemism was reported a century ago.

**New information:**

Based on the fish specimens collected from markets, rivers, swamps, ponds and ditches around Inle Lake as well as from the lake itself from 2014 to 2016, we recorded a total of 948 occurrence data (2120 individuals), belonging to 10 orders, 19 families, 39 genera and 49 species. Amongst them, 13 species of 12 genera are endemic or nearly endemic to the lake system and 17 species of 16 genera are suggested as non-native. The data are all accessible from the document “A dataset of Inle Lake fish fauna and its distribution (http://ipt.pensoft.net/resource.do?r=inle_fish_2014-16)”, as well as DNA barcoding data (mitochondrial COI) for all species being available from the DDBJ/EMBL/GenBank (Accession numbers: LC189568–LC190411). Live photographs of almost all the individuals and CT/3D model data of several specimens are also available at the graphical fish biodiversity database (http://ffish.asia/INLE2016; http://ffish.asia/INLE2016-3D). The information can benefit the clarification, public concern and conservation of the fish biodiversity in the region.

## Introduction

Inle Lake is located on the southwestern part of Shan State, which is the easternmost state of Myanmar. The lake is surrounded by Shan Hills, which isolate it from the neighbouring aquatic habitats. The lake harbours several endemic fish species (Annandale 1918). However, the detailed information of fish fauna of this region has long been unknown since Annandale (1918), while several studies reported the concerns for settlement of non-native species and decline of endemic/native species (Musikasinthorn 1998, Su and Jassby 2000, Davies et al. 2004, Oo 2010). This project aimed to elucidate the current status of fish fauna of the lake, especially focusing on survival of endemic/native species and settlement of non-native species. In addition, DNA sequences (mitochondrial COI), photo images and CT/3D models were published online in the public interest of biodiversity.

## Project description

### Title

Current status, origin and conservation of endemic fishes in an ancient lake, Inle Lake.

### Personnel

Katsutoshi Watanabe (Project director, fieldwork and DNA barcoding), Prachya Musikasinthorn (fieldwork, fish identification, photographs and specimens management), Yuichi Kano (fieldwork, photographs and database management), Akihisa Iwata (fieldwork and fish identification), Shoko Matsui (DNA barcoding), Ryoichi Tabata (DNA barcoding), Sein Tun (management and local information), LKC Yun (fieldwork and local information), Seint Seint Win (fieldwork and local information) and Taksehi Yamasaki (CT scanning).

### Study area description

Inle Lake is located on the Southwestern part of Shan State, which is the easternmost state in Myanmar. The lake is surrounded by Shan Hills and harbours several endemic fish species. The surveys were carried out in the lake and its surroundings from 23 September 2014 until 2 July 2016. In addition, local markets were also investigated for fishes caught in the study area.

### Design description

This study focused on fish fauna of Inle Lake, a representative ancient lake in mainland Southeast Asia. Until now, the lake has not been investigated for nearly a century (Annandale 1918). In our study, we focused on the condition of endemic species as the environments have been drastically changed (Su and Jassby 2000) and alien species recently settled in the lake (Davies et al. 2004, Oo 2010). As information about the fish species of Inle Lake is quite limited, almost all the specimens were photographed and the photo data were deposited in an online fish database (Kano et al. 2013) (http://ffish.asia/INLE2016). CT/3D models for several specimens were also made and are available from the database (http://ffish.asia/INLE2016-3D). The main bodies were retained as formalin samples for voucher specimens and deposited at Kasetsart University, Thailand. In addition, a small piece of tissue (mainly from the right pectoral or pelvic fin) was excised, preserved in 99% alcohol and stored at Kyoto University to facilitate the study of molecular biology and genetics. The DNA sequences (mitochondrial COI) information was deposited at the DDBJ/EMBL/GenBank (Accession numbers: LC189568–LC190411).

### Funding

JSPS (The Japan Society for the Promotion of Science) KAKENHI Grant Number JP26304007.

## Sampling methods

### Sampling description

**Figure 1.**
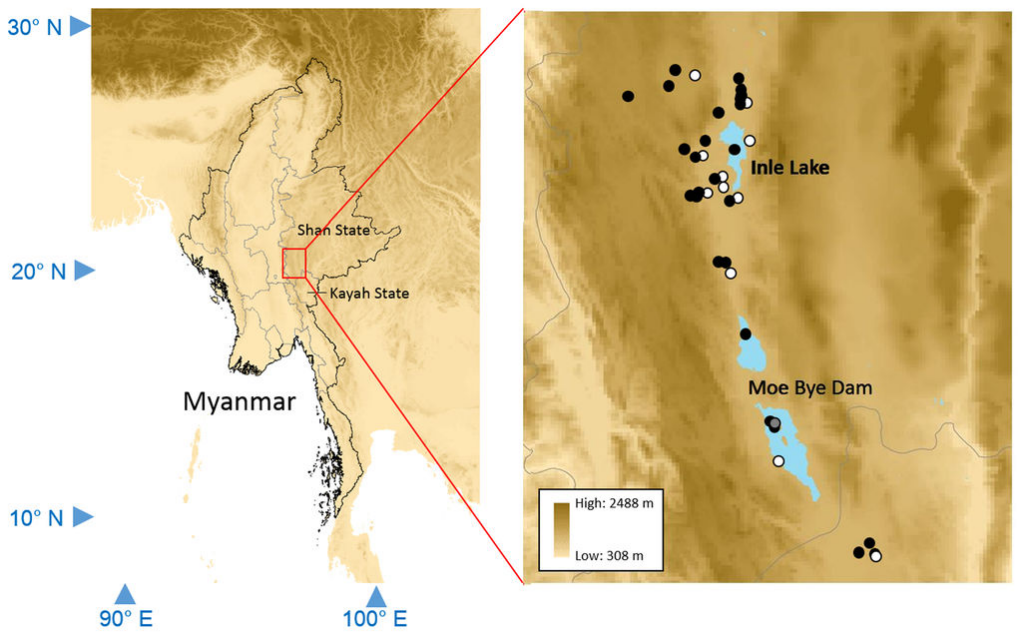
Spatial coverage of the sampling points. Solid circles indicate wild environments such as rivers and reservoirs. White circles indicate local markets and a grey circle indicates a fish cage in the lake.

The fish samples were either collected from 43 wild habitats as well as from one fish cage in the lake or purchased at 24 local markets (Fig. 1​). The fishes were collected by hand-nets, throwing nets, traps and/or purchased from fishermen. In the markets, we avoided purchasing the obvious cultured fishes from other areas (especially from Yangon and Mandalay), such as *Wallago* spp. and *Pangasianodon hypophthalmus*, by checking with the seller.

### Quality control

All the scientific names of fish samples were validated by the updated fish checklist in the Catalogue of Fishes, California Academy of Sciences (http://www.calacademy.org/scientists/projects/catalog-of-fishes), Kottelat (2013) and/or Nelson et al. (2016). For order level classification of the family Ambassidae, we followed Hastings et al. (2014). Most of the specimens were photographed in a fresh state (Kano and Nakajima 2014) and then the formalin specimens and its tissue samples were catalogued and deposited at the Research Laboratory of Ichthyology, Department of Fishery Biology, Faculty of Fisheries, Kasetsart University, Bangkok, Thailand (RLIKU) and Kyoto University, Kyoto, Japan, respectively. All the samples were assigned the IDs which were associated with the records of location (latitude, longitude and region name), the dates, methods, accession numbers of DNA sequences, etc.

### Step description

**Step 1**: Sampling locality and date were recorded.

**Step 2**: Specimens were given IDs, photographed, fin-clipped and roughly classified on site.

**Step 3**: Specimens were fixed in 10% formalin solution for two or more weeks. Subsequently, they were cleaned with water and preserved in 75% ethanol.

**Step 4**: Specimens were shipped back to the lab for correct species identification.

## Taxonomic coverage

### Description

**Figure 2.**
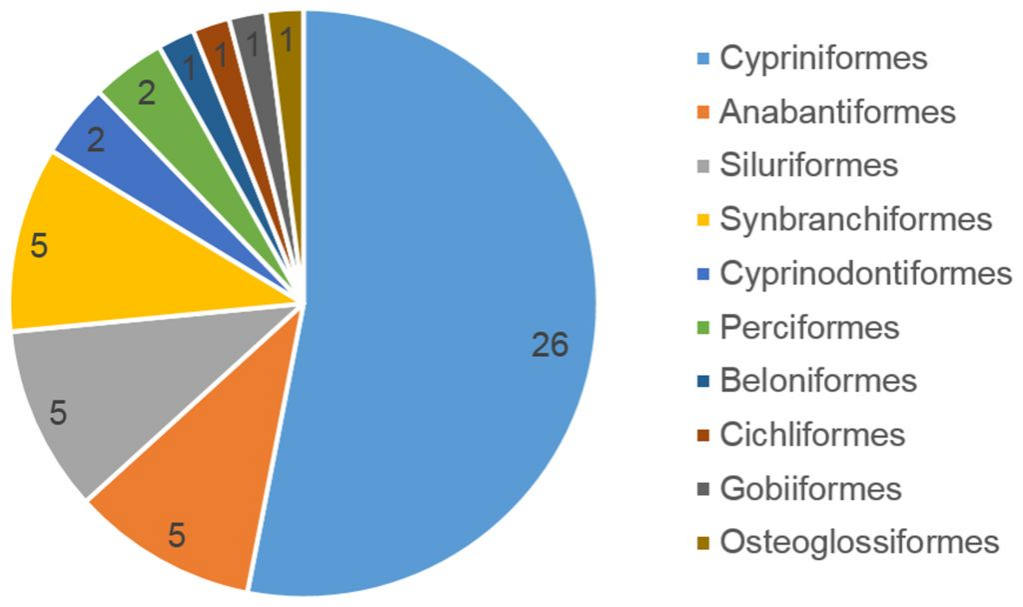
Taxonomic coverage (by order).

**Figure 3.**
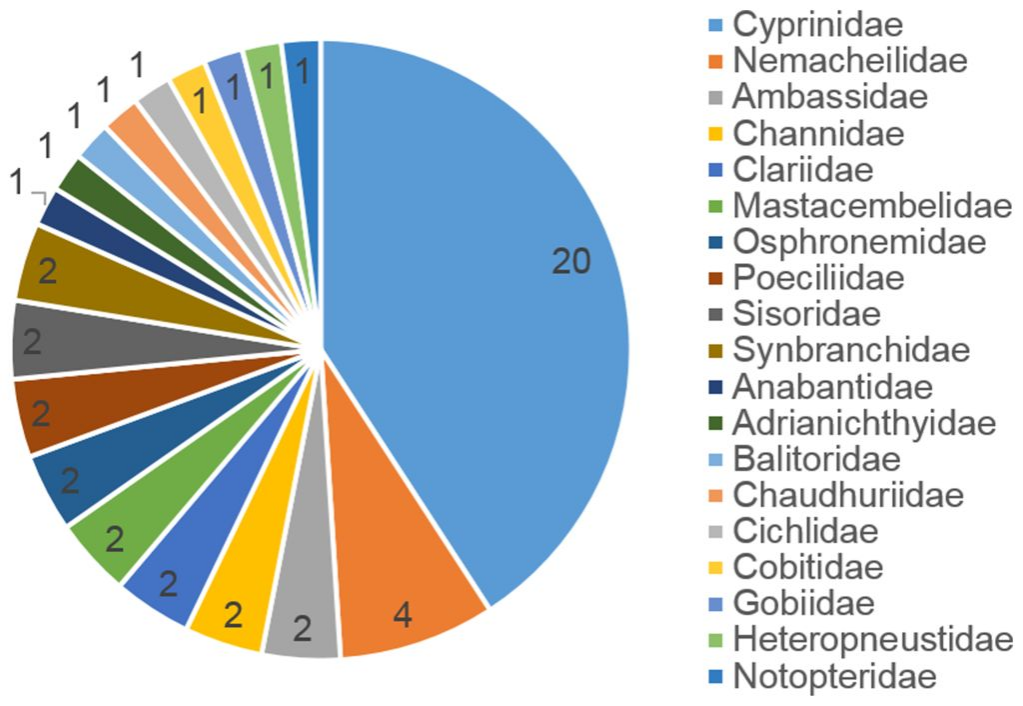
Taxonomic coverage (by family).

Annandale (1918), Akihito and Meguro (1975), Roberts (1986), Kottelat (1990), Talwar and Jhingran (1991), Fang (1997), Roberts (1997), Musikasinthorn (1998), Roberts (1998), Kottelat and Witte (1999), Ng et al. (1999), Kottelat (2001), Fang (2003), Kottelat (2003), Roberts (2007), Ng and Kottelat (2008), Fang et al. (2009), Matsumoto et al. (2009), Britz (2010), Havird and Page (2010), Jiang et al. (2012), Kottelat (2012), Kottelat et al. (2012), Pethiyagoda et al. (2012), Kottelat (2013), Hastings et al. (2014), Ratmuangkhwang et al. (2014) and Nelson et al. (2016) were used as a taxonomic reference for this work. The coverage of this dataset includes Subclass Actinopterygii. The orders are Cypriniformes (26 species), Anabantiformes (5), Siluriformes (5), Synbranchiformes (5), Cyprinodontiformes (2), Perciformes (2), Beloniformes (1), Cichliformes (1), Gobiiformes (1) and Osteoglossiformes (1) (Fig. 2). The families are Cyprinidae (20), Nemacheilidae (4), Ambassidae (2), Channidae (2), Clariidae (2), Mastacembelidae (2), Osphronemidae (2), Poeciliidae (2), Sisoridae (2), Synbranchidae (2), Anabantidae (1), Adrianichthyidae (1), Balitoridae (1), Chaudhuriidae (1), Cichlidae (1), Cobitidae (1), Gobiidae (1), Heteropneustidae (1) and Notopteridae (1) (Fig. 3).

### Taxa included

**Table:** 

Rank	Scientific Name	Common Name
kingdom	Animalia	Animals
phylum	Chordata	Chordates
subphylum	Craniata	Vertebrates and hagfishes
class	Osteichthyes	Bony fishes and tetrapods
subclass	Actinopterygii	Ray-finned fishes
order	Anabantiformes	Labyrinth fishes
order	Beloniformes	Needlefishes
order	Cichliformes	Cichlids and convict blennies
order	Cypriniformes	Carps, loaches, minnows and relatives
order	Cyprinodontiformes	Killifishes
order	Gobiiformes	Gobies
order	Osteoglossiformes	Bonytongues
order	Perciformes	Perches
order	Siluriformes	Catfishes
order	Synbranchiformes	Swamp eels
family	Anabantidae	Climbing gouramies
family	Adrianichthyidae	Adrianichthyids
family	Ambassidae	Asiatic glassfishes
family	Balitoridae	Hillstream loaches
family	Channidae	Snakeheads
family	Chaudhuriidae	Earthworm eels
family	Cichlidae	Cichlids
family	Clariidae	Airbreathing catfishes
family	Cobitidae	True loaches
family	Cyprinidae	Cyprinids
family	Gobiidae	Gobies
family	Heteropneustidae	Airsac catfishes
family	Mastacembelidae	Spiny eels
family	Nemacheilidae	Stone loaches
family	Notopteridae	Knifefishes
family	Osphronemidae	Gouramies and fighting fishes
family	Poeciliidae	Livebearers
family	Sisoridae	Sisorid catfishes
family	Synbranchidae	Swamp eels
species	*Anabas testudineus* (Bloch 1792)	Climbing gourami
species	*Balitora* sp.	A species of balitorid loach
species	*Barbonymus gonionotus* (Bleeker 1849)	Silver barb
species	*Celestichthys erythromicron* (Annandale 1918)	A species of Celestichthys minnow
species	*Channa harcourtbutleri* (Annandale 1918)	Inle snakehead
species	*Channa striata* (Bloch 1793)	Striped snakehead
species	*Chaudhuria caudata* Annandale 1918	Inle swamp eel
species	*Clarias gariepinus* (Burchell 1822)	African sharptooth catfish
species	Clarias cf. batrachus (Linnaeus 1758)	Walking catfish
species	*Ctenopharyngodon idella* (Valenciennes 1844)	Grass carp
species	*Cyprinus intha* Annandale 1918	Inle carp
species	*Cyprinus rubrofuscus* Lacepède 1803	Common carp
species	*Devario kakhienensis* (Anderson 1879)	A species of Devario minnow
species	*Devario* sp.	A species of Devario minnow
species	*Esomus danrica* (Hamilton 1822)	Flying barb
species	*Gambusia affinis* (Baird & Girard 1853)	Western mosquitofish
species	*Garra gravelyi* (Annandale 1919)	Burmese Garra
species	Glossogobius cf. giuris (Hamilton 1822)	A species of Glossogobius goby
species	*Glyptothorax granosus* Jiang, Ng, Yang & Chen 2012	A species of sisorid catfish
species	*Glyptothorax rugimentum* Ng & Kottelat 2008	A species of sisorid catfish
species	*Gymnostomus horai* (Bănărescu 1986)	A species of Gymnostomus minnow
species	*Heteropneustes fossilis* (Bloch 1794)	Stinging catfish
species	*Inlecypris auropurpureus* (Annandale 1918)	A species of Inlecypris minnow
species	*Labeo rohita* (Hamilton 1822)	Rohu
species	*Lepidocephalichthys berdmorei* (Blyth 1860)	A species of cobitid loach
species	*Mastacembelus caudiocellatus* (Boulenger 1893)	A species of spiny eel
species	*Mastacembelus oatesii Boulenger* 1893	A species of spiny eel
species	*Microrasbora rubescens* Annandale 1918	Red dwarf rasbora
species	*Monopterus cuchia* (Hamilton 1822)	Gangetic mud eel
species	*Monopterus javanensis* Lacepède 1800	Asian swamp eel
species	*Neolissochilus nigrovittatus* (Boulenger 1893)	A species of Neolissochilus barb
species	*Notopterus notopterus* (Pallas 1769)	Bronze featherback
species	*Oreochromis niloticus* (Linnaeus 1758)	Nile tilapia
species	*Oryzias uwai* Roberts 1998	A species of rice fish
species	*Parambassis lala* (Hamilton 1822)	A species of Asiatic glassfish
species	*Parambassis ranga* (Hamilton 1822)	A species of Asiatic glassfish
species	*Pethia stoliczkana* (Day 1870)	Stoliczka's barb
species	*Petruichthys brevis* (Boulenger 1893)	A species of nemacheilid loach
species	*Physoschistura rivulicola* (Hora 1929)	A species of nemacheilid loach
species	*Physoschistura shanensis* (Hora 1929)	A species of nemacheilid loach
species	*Poecilia reticulata* Peters 1859	Guppy
species	*Poropuntius schanicus* (Boulenger 1893)	A species of Poropuntius barb
species	*Puntius sophore* (Hamilton 1822)	Spotfin swamp barb
species	Puntius cf. sophore (Hamilton 1822)	A species of Puntius barb
species	*Sawbwa resplendens* Annandale 1918	Burmese rammy nose
species	*Schistura* sp.	A species of nemacheilid loach
species	Systomus cf. rubripinnis (Valenciennes 1842)	A species of Systomus barb
species	*Trichogaster labiosa* Day 1877	Thick-lipped gourami
species	*Trichopodus pectoralis* Regan 1910	Snakeskin gourami

## Temporal coverage

### Notes

23 September 2014 – 2 July 2016.

## Usage rights

### Use license

Creative Commons Public Domain Waiver (CC-Zero)

## Data resources

### Data package title

A Dataset of Inle Lake Fish Fauna and Its Distribution

### Resource link

http://ipt.pensoft.net/resource.do?r=inle_fish_2014-16

### Number of data sets

1

## Additional information

### Endemic, native and non-native or status uncertain

**Figure 4.**
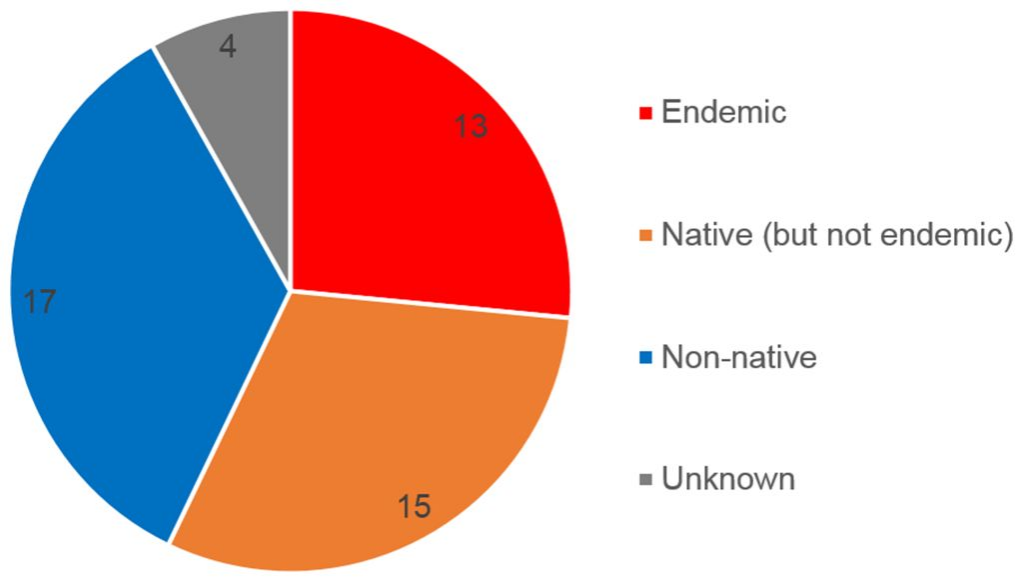
Distribution of the endemic, native, non-native and uncertain species in the studied area.

Inle Lake has an outstanding endemic fish fauna while non-native species have established in and around the lake. Thus, the species were discriminated by endemic, native (but not endemic), non-native and unknown as shown in Fig. 4. In addition, two endemic species reported in Annandale (1918) were not ascertained in this survey: the two species seemed to be very rare or already extinct from the studied area.

**Endemic**: *Celestichthys erythromicron*; *Channa harcourtbutleri*; *Cyprinus intha*; *Gymnostomus horai*; *Inlecypris auropurpureus*; *Mastacembelus caudiocellatus*; *Mastacembelus oatesii*; *Microrasbora rubescens*; *Neolissochilus nigrovittatus*; *Petruichthys brevis*; *Physoschistura shanensis; Poropuntius schanicus*; *Sawbwa resplendens*.

**Native (but not endemic)**: *Anabas testudineus*; *Channa striata*; *Chaudhuria caudata*; Clarias cf. batrachus; *Devario kakhienensis*; *Garra gravelyi*; *Glyptothorax granosus*; *Glyptothorax rugimentum*; *Lepidocephalichthys berdmorei*; *Monopterus cuchia*; *Monopterus javanensis*; *Notopterus notopterus*; *Pethia stoliczkana*; *Physoschistura rivulicola*; Systomus cf. rubripinnis.

**Non-native**: *Barbonymus gonionotus*; *Clarias gariepinus*; *Ctenopharyngodon idella*; *Cyprinus rubrofuscus*; *Esomus danrica*; *Gambusia affinis*; Glossogobius cf. giuris; *Heteropneustes fossilis*; *Labeo rohita*; *Oreochromis niloticus*; *Oryzias uwai*; *Parambassis lala*; *Parambassis ranga*; *Poecilia reticulata*; *Puntius sophore*; *Trichogaster labiosa*; *Trichopodus pectoralis*.

**Unknown**: *Balitora* sp.; *Devario* sp.; Puntius cf. sophore; *Schistura* sp.

**Endemic species unascertained**: *Systomus compressiformis*; *Silurus burmanensis*.

## References

[B3405238] Akihito Prince, Meguro K. (1975). Description of a new gobiid fish, *Glossogobius
aureus*, with notes on related species of the genus. Japanese Journal of Ichthyology.

[B3405248] Annandale N. (1918). Fish and fisheries of the Inle Lake. Records of the Indian Museum.

[B3414314] Britz R. (2010). Species of the *Macrognathus
aculeatus* group in Myanmar with remarks on *Mastacembelus
caudiocellatus* (Teleostei: Synbranchiformes: Mastacembelidae). Ichthyological Exploration of Freshwaters.

[B3405731] Davies J., Sebastian A. C., Chan S. (2004). A wetland inventory for Myanmar.

[B3414333] Fang F. (1997). Redescription of *Danio
kakhienensis*, a poorly known cyprinid fish from the Irrawaddy basin. Ichthyological Exploration of Freshwaters.

[B3405258] Fang Fang (2003). Phylogenetic analysis of the Asian cyprinid genus *Danio* (Teleostei, Cyprinidae). Copeia.

[B3405269] Fang Fang, Noren Michael, Liao Te Yu, Källersjö Mari, Kullander S. O. (2009). Molecular phylogenetic interrelationships of the south Asian cyprinid genera *Danio*, *Devario* and *Microrasbora* (Teleostei, Cyprinidae, Danioninae). Zoologica Scripta.

[B3407476] Hastings Philip A., Walker Harold Jack, Galland Grantly R. (2014). Fishes : a guide to their diversity.

[B3405291] Havird Justin C., Page Lawrence M. (2010). A revision of *Lepidocephalichthys* (Teleostei: Cobitidae) with descriptions of two new species from Thailand, Laos, Vietnam, and Myanmar. Copeia.

[B3405301] Jiang WANSHENG, Ng HEOK HEE, Yang JUNXING, Chen XIAOYONG (2012). A taxonomic review of the catfish identified as *Glyptothorax
zanaensis* (Teleostei: Siluriformes: Sisoridae), with the descriptions of two new species. Zoological Journal of the Linnean Society.

[B3405337] Kano Yuichi, Nakajima Jun (2014). Non-killing simple photography techniques of small–middle freshwater fishes in the field. Japanese Journal of Ichthyology.

[B3405311] Kano Yuichi, Adnan Mohad Shalahuddin, Grudpan Chaiwut, Grudpan Jarungjit, Magtoon Wichan, Musikasinthorn Prachya, Natori Yoshihiro, Ottomanski Stefan, Praxaysonbath Bounthob, Phongsa Koneouma, Rangsiruji Achariya, Shibukawa Koichi, Shimatani Yukihiro, So Nam, Suvarnaraksha Apinun, Thach Phanara, Thanh Phuong Nguyen, Tran Dac Dinh, Utsugi Kenzo, Yamashita Tomomi (2013). An online database on freshwater fish diversity and distribution in Mainland Southeast Asia. Ichthyological Research.

[B3405356] Kottelat M. (1990). Indochinese nemacheilines: A revision of nemacheiline loaches (Pisces: Cypriniformes) of Thailand, Burma, Laos, Cambodia and southern Viet Nam.

[B3414324] Kottelat M. (2001). Fishes of Laos.

[B3405365] Kottelat Maurice (2003). Nomenclatural status of *Crossocheilus
burmanicus*, *Crossocheilus
horai* and *Crossocheilus
multirastellatus* (Osteichthyes: Cyprinidae). Raffles Bulletin of Zoology.

[B3405375] Kottelat M. (2012). Conspectus cobitidum: an inventory of the loaches of the world (Teleostei: Cypriniformes: Cobitoidei). The Raffles Bulletin of Zoology.

[B3405402] Kottelat M (2013). The fishes of inland waters of Southeast Asia: A catalogue and core bibliography of fishes known to occur in freshwaters, mangroves and estuaries. The Raffles Bulletin of Zoology.

[B3405412] Kottelat M,, Witte K. E. (1999). Two new species of *Microrasbora* from Thailand and Myanmar, with two new generic names for small Southeast Asian cyprinid fishes (Teleostei: Cyprinidae). Journal of South Asian Natural History.

[B3405385] Kottelat M, Baird IG, Kullander S. O., Ng HH, Parenti LR, Rainboth WJ, Vidthayanon C, Allen DJ, Smith KG, Darwall WRT (2012). The status and distribution of freshwater fishes of Indo-Burma. The status and distribution of freshwater biodiversity in Indo-Burma.

[B3405422] Matsumoto Seiji, Kon Takeshi, Yamaguchi Motoomi, Takeshima Hirohiko, Yamazaki Yuji, Mukai Takahiko, Kuriiwa Kaoru, Kohda Masanori, Nishida Mutsumi (2009). Cryptic diversification of the swamp eel *Monopterus
albus* in East and Southeast Asia, with special reference to the Ryukyuan populations. Ichthyological Research.

[B3405437] Musikasinthorn P (1998). First record of *Parambassis
lala* (Pisces: Ambassidae) from Inle Lake, the Salween River Basin, Myanmar. Natural History Bulletin of the Siam Society.

[B3406242] Nelson Joseph S., Grande Terry C., Wilson Mark V. H. (2016). Fishes of the world (5th edition).

[B3405467] Ng Heok H., Ng Peter K. L., Britz Ralf (1999). *Channa
harcourtbutleri* (Annandale, 1918): a valid species of snakehead (Perciformes: Channidae) from Myanmar. Journal of South Asian Natural History.

[B3405447] Ng HEOK HEE, Kottelat MAURICE (2008). The identity of *Clarias
batrachus* (Linnaeus, 1758), with the designation of a neotype (Teleostei: Clariidae). Zoological Journal of the Linnean Society.

[B3405477] Oo A. H., Weimin M., Silva S. D., Davy B. (2010). Inland fisheries resources enhancement and conservation practice in Myanmar. Inland fisheries resource enhancement and conservation in Asia.

[B3405501] Pethiyagoda R, Meegaskumbura M, Maduwage K (2012). A synopsis of the South Asian fishes referred to *Puntius* (Pisces: Cyprinidae). Ichthyological Exploration of Freshwaters.

[B3405511] Ratmuangkhwang Sahat, Musikasinthorn Prachya, Kumazawa Yoshinori (2014). Molecular phylogeny and biogeography of air sac catfishes of the *Heteropneustes
fossilis* species complex (Siluriformes: Heteropneustidae). Molecular Phylogenetics and Evolution.

[B3405521] Roberts T. R. (1986). Systematic review of the Mastacembelidae or spiny eels of Burma and Thailand, with description of two new species of *Macrognathus*. Japanese Journal of Ichthyology.

[B3414304] Roberts T. R. (1997). Systematic revision of the tropical Asian labeoin cyprinid fish genus *Cirrhinus*, with descriptions of new species and biological observations on *Cirrhinus
lobatus*. Natural History Bulletin of the Siam Society.

[B3405531] Roberts T. R. (1998). Systematic observations on tropical Asian medakas or ricefishes of the genus *Oryzias*, with descriptions of four new species. Ichthyological Research.

[B3405541] Roberts T. R. (2007). The "celestial pearl danio", a new genus and species of colourful minute cyprinid fish from Myanmar (Pisces: Cypriniformes). The Raffles Bulletin of Zoology.

[B3405551] Su Myint, Jassby Alan D. (2000). Inle: a large Myanmar lake in transition. Lakes and Reservoirs: Research and Management.

[B3405561] Talwar P. K., Jhingran A. G. (1991). Inland fishes of India and adjacent countries. Vol. 1–2.

